# Heart Rate Variability and Cognition: A Narrative Systematic Review of Longitudinal Studies

**DOI:** 10.3390/jcm13010280

**Published:** 2024-01-04

**Authors:** Paola Nicolini, Gabriella Malfatto, Tiziano Lucchi

**Affiliations:** 1 Fondazione IRCCS Ca’ Granda Ospedale Maggiore Policlinico, Geriatric Unit, Internal Medicine Department, 20122 Milan, Italy; tiziano.lucchi@policlinico.mi.it; 2 Istituto Auxologico Italiano IRCCS, Department of Cardiovascular, Neural and Metabolic Sciences, Ospedale San Luca, 20149 Milan, Italy; malfi@auxologico.it

**Keywords:** heart rate variability, cognition, longitudinal studies, systematic review, autonomic nervous system

## Abstract

Background: Heart rate variability (HRV) is a reliable and convenient method to assess autonomic function. Cross-sectional studies have established a link between HRV and cognition. Longitudinal studies are an emerging area of research with important clinical implications in terms of the predictive value of HRV for future cognition and in terms of the potential causal relationship between HRV and cognition. However, they have not yet been the objective of a systematic review. Therefore, the aim of this systematic review was to investigate the association between HRV and cognition in longitudinal studies. Methods: The review was conducted according to the Preferred Reporting Items for Systematic Reviews and Meta-Analyses (PRISMA) guidelines. The Embase, PsycINFO and PubMed databases were searched from the earliest available date to 26 June 2023. Studies were included if they involved adult human subjects and evaluated the longitudinal association between HRV and cognition. The risk of bias was assessed with the Newcastle–Ottawa Scale for Cohort Studies. The results were presented narratively. Results: Of 14,359 records screened, 12 studies were included in this systematic review, with a total of 24,390 participants. Two thirds of the studies were published from 2020 onwards. All studies found a longitudinal relationship between HRV and cognition. There was a consistent association between higher parasympathetic nervous system (PNS) activity and better cognition, and some association between higher sympathetic nervous system activity and worse cognition. Also, higher PNS activity persistently predicted better executive functioning, while data on episodic memory and language were more scant and/or controversial. Conclusions: Our results support the role of HRV as a biomarker of future cognition and, potentially, as a therapeutic target to improve cognition. They will need confirmation by further, more comprehensive studies also including unequivocal non-HRV sympathetic measures and meta-analyses.

## 1. Introduction

### 1.1. Heart Rate Variability

Heart rate variability (HRV) is a physiological phenomenon characterized by fluctuations in the time intervals between consecutive heartbeats and it reflects the influence on the sinus node of the two limbs of the autonomic nervous system (ANS)—sympathetic (SNS) and parasympathetic (PNS) [1,2,3]. HRV analysis is thus a simple, non-invasive and reliable method of assessing autonomic function in the most diverse areas of clinical practice [4,5,6]. HRV analysis is traditionally performed in the time and frequency domains [1,2,3], but other measures can also be computed, including heart rate fragmentation (HRF) metrics [7,8] and non-linear indices [3,9,10].

Time-domain indices derive from simple statistical processing of the inter-beat intervals, and the most popular are the standard deviation of the normal-to-normal (NN) intervals (SDNN), the root mean square of successive differences of the NN intervals (RMSSD) and the percentage of successive NN intervals that differ by more than 50 ms (pNN50) [1,2,3].

Frequency-domain indices result from the decomposition of the signal into different frequency bands, most commonly by fast Fourier transform (FFT) or autoregressive (AR) modeling: total power (TP, ≤0.4 Hz), ultra-low frequency power (≤0.003 Hz, only in 24-h recordings), very low-frequency power (VLF, 0.003–0.04 Hz), low-frequency power (LF, 0.04–0.15 Hz) and high-frequency power (HF, 0.15–0.4 Hz) [1,2,3]. Based on LF and HF, transformed indices can also be calculated: normalized LF [LFn = LF/(LF + HF) × 100], normalized HF [HFn = HF/(LF + HF) × 100] and the LF/HF ratio [1,2,3].

HRF metrics quantify the fragmentation of the heart rhythm which is manifested by abrupt changes in the sign of the HR (from acceleration to deceleration and vice versa) [7,8]. They are statistical metrics that comprise the percentage of inflection points (PIP), the percentage of ∆ NN intervals in long segments (PNNLS) and the percentage of ∆ NN intervals in short segments (PNNSS). The greater the HRF, the higher PIP and PNNSS and the lower PNNLS [7,8].

Non-linear indices (e.g., Poincaré plot parameters, entropy, detrended fluctuation analysis coefficients) [3,9,10] were not used by the studies included in this review and are thus briefly addressed in the Discussion.

The different indices differ in terms of physiological significance. RMSSD, pNN50 and HF are parasympathetic indices [1,2,3]. SDNN and TP reflect joint sympathetic and parasympathetic modulation but can be taken as primarily parasympathetic indices in resting conditions, when vagal tone prevails [1,2,3]. The nature of LF is highly controversial, and it has been viewed as an index of prevalently sympathetic modulation [1], mixed sympathetic and parasympathetic modulation [1,11], and predominantly parasympathetic modulation [12]. ULF and VLF have uncertain physiological correlates [1,3]. LFn and LF/HF have been considered sympathetic indices (and HFn a parasympathetic index) by many authors (e.g., [13,14,15,16,17]). However, in the HRV literature, the use of transformed indices as markers of SNS activity is a matter of ongoing debate (e.g., [18]), especially in relation to the LF/HF ratio (e.g., [19,20,21]). The physiological underpinnings of HRF are still unresolved, but it is believed to reflect a degradation of the parasympathetic nervous system, such that increased HRF is a marker of abnormally decreased vagal activity [7].

### 1.2. Heart Rate Variability and Cognition

An increasing number of cross-sectional studies have shown there is an association between HRV and cognition, in generally healthy individuals across the age spectrum (for reviews, see [22,23]) as well as in subjects with neurocognitive disorders (for reviews see [23,24,25,26]) and with neuropsychiatric conditions (e.g., [27,28,29,30]). There are several possible explanations for this link.

First, HRV and cognition share a common neural substrate which is the central autonomic network (CAN) [31,32,33]. The CAN is a complex system of brain regions that are implicated both in cognitive processing and in the autonomic modulation of cardiovascular function via projections to the preganglionic neurons of the SNS and PNS [31,32,33]. It therefore represents the neuroanatomical correlate of the brain–heart axis [32,33].

Second, the ANS controls cerebral blood flow through an indirect and a direct mechanism. The former is the regulation of blood pressure (BP), whether in terms of absolute BP values [34] or BP variability (BPV) [35,36]. The latter is the autonomic innervation of the cerebral vasculature [37]. Therefore, autonomic dysfunction can lead to cerebral hypoperfusion, brain damage and cognitive impairment [36,37,38,39].

Third, the ANS plays a role in inflammation. In particular, there exists a cholinergic anti-inflammatory pathway (CAP) by which the release of acetylcholine by the vagus nerve acts on splenic macrophages to inhibit the synthesis of pro-inflammatory cytokines [40,41]. Thus, reduced vagal activity is linked to systemic inflammation which can propagate to the central nervous system (i.e., neuroinflammation) to induce pathogenic brain changes [42,43,44] and cognitive decline [45,46].

Over the last decade, there has been an exponential rise in the number of publications on HRV and cognition [23]. Although the bulk of studies remain cross-sectional [22,47], longitudinal studies are beginning to emerge.

The clinical relevance of longitudinal studies lies in the fact that they alone can evaluate whether HRV can predict future cognitive performance. This means that, within the heterogeneous trajectories of cognitive aging [48,49], HRV could serve as a potential biomarker to identify high-risk subjects to whom interventions should be directed. Also, longitudinal studies support causal inference [50] and can help establish if autonomic dysfunction causes cognitive impairment. In this case, HRV could become a valuable therapeutic target since treatments to improve cognition are limited by poor efficacy and drug side-effects [51].

Therefore, the aim of this systematic review was to search the literature for longitudinal studies investigating the association between HRV and cognition, to extract data on the study characteristics, and to summarize and discuss the available evidence. Among the study characteristics, we also focused on HRV methodology since a standardized approach to HRV assessment according to recognized guidelines [1,2] is essential to ensure valid interpretation of HRV measures and their comparability across studies.

To the best of our knowledge, there is no other review specifically addressing the longitudinal relationship between HRV and cognition. The latest reviews on HRV and cognition that include both longitudinal and cross-sectional studies contain very few of the former. The systematic review by Forte et al. [22], restricted to healthy subjects and including studies up to 2018, covers three longitudinal studies. A very recent narrative review by Arakaki et al. [23], likely due to the breadth of its scope, encompasses only two longitudinal studies.

## 2. Materials and Methods

The review process was conducted according to the Preferred Reporting Items for Systematic Reviews and Meta-Analyses (PRISMA) guidelines [52] by two independent authors (P.N. and G.M.). Any areas of uncertainty or disagreement were adjudicated by a third author (T.L.). The review was not registered.

### 2.1. Literature Search

We performed an electronic literature search of the Embase, PsycINFO and PubMed databases from the earliest available date to 26 June 2023. We used the following search terms: (“heart rate variability” OR “HRV” OR “autonomic” OR “vagal” OR “parasympathetic” OR “sympathetic”) AND (“cognit*” OR “neuropsych*” OR “neurocognit*” OR “dementia”) without filters or limits. Although the review focused on longitudinal studies, in accordance with other authors (e.g., [53,54]), in order to avoid the search being too restrictive, the study design (i.e., “longitudinal”) was not included in the search terms, but rather in the inclusion/exclusion criteria.

### 2.2. Inclusion/Exclusion Criteria

Studies were included if they were carried out on adult (aged ≥ 18) human subjects and investigated the association between HRV (exposure) and cognition (outcome) in a longitudinal study design. We adopted a broad definition of longitudinal study [55], i.e., one with at least a baseline HRV assessment and a follow-up cognitive evaluation. We considered all HRV measures and all cognitive outcomes (performance on tests of global and domain-specific cognition, incidence of cognitive impairment). Only original research articles, published in English with available full text, were included. We excluded intervention studies and studies examining non-HRV autonomic function (e.g., by cardiovascular autonomic tests and autonomic symptom scales). We also excluded reviews, meta-analyses, case reports/series, editorials, letters, commentaries, book chapters, conference proceedings and dissertations.

### 2.3. Data Extraction

The following information was extracted from the included studies: (1) first author and year of publication, (2) study design (i.e., strictly longitudinal or longitudinal time-lagged), (3) sample size, (4) characteristics of the participants at baseline including type of population (general versus clinical) and demographics (age, sex, education, ethnicity), (5) study exclusion criteria (in terms of comorbidities and medications), (6) duration of follow-up, (7) HRV methodology, including HRV recording device, conditions and length of the recording, type of HRV indices and frequency-domain analysis method used, stabilization period, artifact correction and assessment of respiration, (8) evaluation of cognition, (9) confounders controlled for in the statistical analyses and (10) study results.

The study design was classified as strictly longitudinal if it met a narrower definition of longitudinal study [55] by measuring HRV at baseline and cognition at baseline and follow-up, or by measuring HRV and cognition both at baseline and follow-up, thus enabling the assessment of across-time cognitive change. It was classified as longitudinal time-lagged if it met a wider definition of longitudinal study [55] by measuring HRV (the antecedent) at baseline and cognition (the outcome) at follow-up.

Given the importance of HRV methodology and the fact that most studies were large-scale epidemiological ones, if specific details of HRV methodology were not directly mentioned in the original article they were retrieved, when possible, from study protocol documentation or related publications from the same wave of the study. This is indicated by a superscript letter in the relevant Tables (Table 1 and Supplementary Table S1, superscript letters c and a respectively) and the additional references used for each study are provided in Supplementary Table S2 [56,57,58,59,60,61,62,63,64,65].

### 2.4. Quality Assessment and Risk of Bias

The methodological quality of the included studies was assessed with the Newcastle–Ottawa Scale for Cohort Studies [76] (see Supplementary Table S3). The Newcastle–Ottawa Scale evaluates three study categories (selection, comparability and outcome) across eight items, and high-quality items are awarded stars (a maximum of one star for all items except for comparability, which can receive two) so that the total score ranges from 0 to 9 stars. The higher the score, the higher the quality of the study and the lower the risk of bias. The results of the Newcastle–Ottawa Scale were converted to the Agency for Health Research and Quality (AHRQ) standards—good quality/low risk of bias, fair quality/moderate risk of bias, poor quality/high risk of bias—based on standard thresholds [77].

### 2.5. Data Synthesis

The data were presented narratively in text and table form, with descriptive statistics as appropriate (e.g., mean, median, range, count and percentage). The study results were grouped in terms of the activity of the specific limbs of the ANS (PNS and SNS) in relation to all cognitive outcomes as well as in relation to specific cognitive domains. Due to study heterogeneity, evaluated based on clinical judgement, a meta-analysis was not performed (see Limitations).

## 3. Results

**Figure 1:**
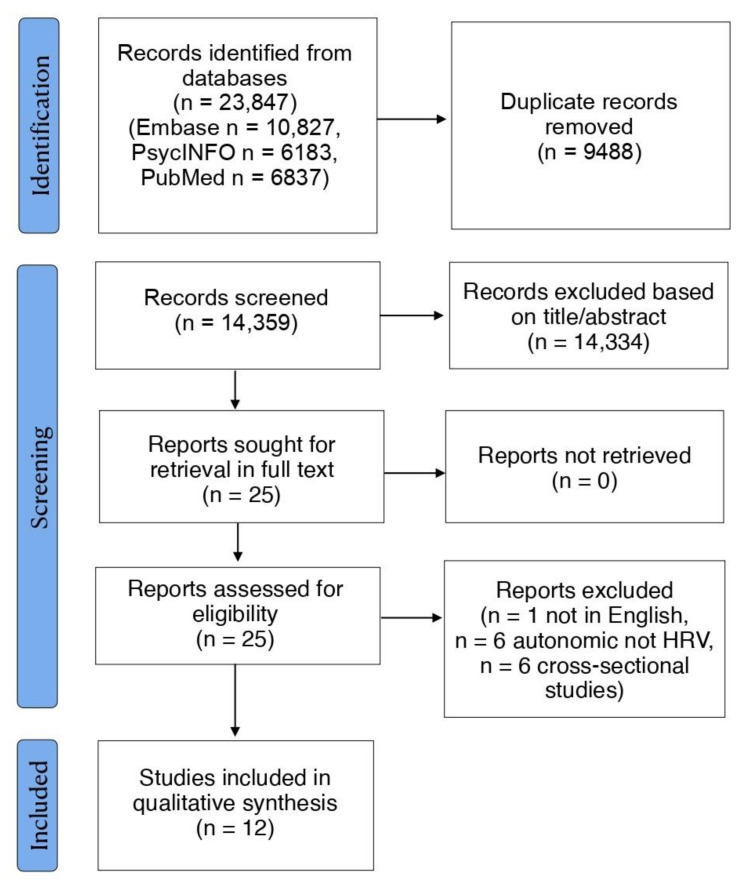
Preferred Reporting Items for Systematic Reviews and Meta-Analyses (PRISMA) flow-diagram.

The literature search identified 14,359 records after duplicates were removed. Following title and abstract screening, 25 articles were retrieved in full-text and screened for eligibility. Of these, 13 were excluded and 12 were examined in detail and included in the current review. The PRISMA flow-diagram is illustrated in Figure 1. An overview of the main features of the included studies is presented in Table 1. Further aspects of HRV methodology across studies can be found in Supplementary Table S1.

### 3.1. Study Publication Year, Design, Sample Size, Participants and Follow-Up

The publication time frame spanned 14 years, from 2008 [66] to 2022 [72,73,74,75] with two thirds of studies clustered in the present decade.

Almost all studies (9 out of 12, 75%) were strictly longitudinal [55], since they assessed HRV at baseline, and cognition at both baseline and follow-up. Among these, Britton et al. [66] also assessed HRV at follow-up but did not evaluate change in HRV. Three studies [64,70,73] were longitudinal time-lagged [55] in that they assessed HRV at baseline and cognition at follow-up. Among these, Schaich et al. [70] also assessed HRV at follow-up.

The number of participants ranged from 71 [74] to 5375 [66]. However, in two thirds of the studies the sample size was in the order of thousands. This reflects the predominance within this field of research of large-scale epidemiological studies. These mainly involved the general population [7,64,66,69,70,71,72,73] but also specific patient populations with or at high risk of vascular disease [67] and with obstructive sleep apnea [75]. Only two studies [68,74] were small-sized non-epidemiological studies in clinical samples with cognitive impairment.

The study populations were highly heterogeneous, including British civil servants [66], subjects enrolled in a statin trial [67] and the adult offspring of the Framingham Heart Study [71]. The mean age at baseline was between 45 [64,73] and 78 [74] years (on average 59.6 years). Indeed, all subjects were either middle-aged [64,66,69,73], middle-aged to older [70,71,72] or older [7,67,68,74,75]. The percentage of females was between 29% [66] and 78% [74]. The level of education varied across studies from a minimum of 8 years [68] to a maximum of 15 to 16 years [73] and from a minimum of less than high school education in about 70% of the sample [75] to a maximum of more than high school education in about 70% of the sample [7]. Three studies did not report education data [66,69,72]. The prevalence of white ethnicity was between 36% [7] and 81% [69]. More than half of studies (7 out of 12, 58%) did not report information on the ethnic composition of the sample.

The duration of follow-up ranged from a minimum of 30 months [68] to a maximum of 16 years [72]. In two studies [71,72] there was a time interval between baseline and the start of the follow-up (5 years and a mean of 14 years, respectively).

### 3.2. Study Exclusion Criteria (Comorbidities and Medications)

Since HRV analysis is by definition performed on sinus beats, several studies (7 out of 12, 58%) explicitly reported excluding participants with arrhythmias (e.g., atrial fibrillation, ectopic atrial and ventricular beats) and/or cardiac pacemakers. Many studies (8 out of 12, 67%) excluded other different comorbidities. Among these, the most common were cardiovascular diseases such as myocardial infarction, stroke/transient ischemic attack (TIA) and heart failure (5 studies out of 8, 63%) and prevalent dementia (4 studies out of 8, 50%). Other comorbidities included diabetes mellitus (3 studies out of 8, 38%), severe liver/kidney/lung diseases (3 studies out of 8, 38%) Parkinson’s disease (2 studies out of 8, 25%) and malignancy (2 studies out of 8, 25%). Less than half of the studies (5 out of 12, 42%) excluded medications. Understandably, the small clinical studies [68,74] were more restrictive in terms of permitted medications than the large-scale epidemiological ones.

### 3.3. HRV Methodology

In all studies but one the HRV recording device was an electrocardiogram (ECG); only Sabil et al. [75] measured HRV through photoplethysmography by employing a pulse oximeter.

Nearly all studies (11 out of 12, 92%) measured HRV in resting conditions, almost always in the supine position except for Knight et al. [69] who used a sitting position. A single study [71] relied on an ambulatory ECG. Only few studies measured HRV in response to a physical or psychological challenge: the former included active standing [69,74] and paced breathing at 12 breaths/min [74], the latter was a cognitive task [69]. The recording was conducted in the morning in most studies (7 out of 12, 58%), during the night in the two sleep studies [7,75] and in an undefined time period in the remaining three studies. In the two small clinical studies [68,74] the time range was restricted to a 3–4 h interval. In half of the studies the participants were instructed to abstain from some combination of smoking (5 studies), caffeinated beverages (4 studies), alcohol (3 studies) or intense physical activity (3 studies) for up to 12 h [74] prior to the recording.

Most studies used short-term ECG recordings: one third used a standard 10-s ECG [64,67,70,73], three studies exclusively used a 5-min ECG [66,72,74] and Knight et al. [69] used both 5- and 10-min ECGs. Some studies used longer-term ECG recordings: Weinstein et al. [71] used 2-h Holter monitoring, Costa et al. [7] and Sabil et al. [75] analyzed HR data from a sleep study with Costa et al. [7] reporting a median duration of sleep of 6 h. One study [68] did not specify the duration of the recording which, however, appeared to be short-term.

All studies measured traditional time- and frequency-domain indices. Costa et al. [7] also computed three HRF metrics which were the mainstay of their analysis. Overall, HRV was most often quantified by time-domain indices, with five studies [64,67,70,71,73] reporting only time-domain indices, one study [69] reporting only a frequency-domain index and six [7,66,68,72,74,75] reporting both. Among the time-domain indices the most common were SDNN (11 studies out of 11) and RMSSD (8 studies out of 11, 73%), while pNN50 was used by only one study.

Frequency-domain analysis was mainly performed by the FFT [68,69,72,74] or the FFT-based Blackman–Tukey method [66]. Only Costa et al. [7] employed the Lomb periodogram. Information on the method of analysis was not available in the case of Sabil et al. [75]. Among the frequency-domain indices, the most popular was HF (6 studies out of 7, 86%), followed by LF (4 studies out of 7, 57%) and the LF/HF ratio (4 studies out of 7, 57%). LFn (2 studies out of 7, 29%), HFn (1 study out of 7, 14%) and TP (2 studies out of 7, 29%) were less common.

Only four studies had a stabilization period which amounted to at least 15 min for Chou et al. [72], 5 or 10 min according to the stage of the experimental protocol for Nicolini et al. [74], and 5 min and at least 5 min for Britton et al. [66] and Schaich et al. [70], respectively.

The artifact detection method was a combination of software-based processing and visual inspection for over half of the studies (7 out of 12, 58%), a fully automated algorithm in three studies, and could not be determined in two studies. In terms of artifact correction, few studies (3 out of 12, 25%) selected artifact-free data, almost half (5 out of 12, 42%) set a threshold of ectopic beats beyond which the recording was excluded from analysis, and for the remaining (4 out of 12, 33%) this information was not accessible. The cut-off for inclusion of ectopics ranged from less than 1% [74] to less than 50% [70]. Data on the technique of artifact correction (specifically interpolation) was available for two studies [69,74].

Although four studies assessed respiration [7,68,74,75] and one [69] had potential access to respiratory data [58], only Nicolini et al. [74] reported the respiratory rate in order to account for its possible confounding effect on HRV analysis (see last paragraph in Section 4.4.3.)

### 3.4. Evaluation of Cognition

Cognition was evaluated by cognitive tests in two thirds of the studies [7,64,66,67,69,70,73,74] and in terms of incidence of dementia in the remaining third [68,71,72,75].

The cognitive tests ranged from 3 [64,67,70] to 13 [74], with most studies (7 out of 8, 88%) employing 6 cognitive tests or fewer. The cognitive batteries covered global cognition (4 studies out of 8, 50%) and different cognitive domains including executive functioning (all studies), episodic memory (6 studies out of 8, 75%) and language (2 studies out of 8, 25%). In terms of the composition of the study batteries, the ratio of episodic memory to executive functioning cognitive tests was between 2:11 [74] and 1:2 [64,67]. Also, only one study [74] assessed both verbal and visual components of episodic memory. Two studies [69,74] computed composite test scores for episodic memory and executive functioning. The cognitive tests used to evaluate global and domain-specific cognition are shown in Table 2 and are in accordance with the standard neuropsychological literature [78,79,80,81,82,83,84,85]. As can be seen, the two language tests also gauged executive functioning due to the close relationship between these two domains [86]. Moreover, of the 20 cognitive tests used across eight studies, over a half were used by a single study (11 out of 20, 55%), less than a half were used by two studies (8 out of 20, 40%), and only one was used by three studies (1 out of 20, 5%).

Incident dementia was identified either through linkage with a national administrative healthcare database [72,75] or via an ad hoc on-site diagnostic process [68,71]. Three studies [71,72,75] considered all-cause dementia, while Kim et al. [68] diagnosed both Alzheimer’s disease (AD) and dementia with Lewy bodies (DLB). The diagnosis of dementia was based on International Classification of Diseases (ICD) codes [72,75] or on established diagnostic criteria [68,71].

### 3.5. Confounders Controlled for in the Statistical Analyses

All studies controlled for demographics (e.g., age, sex, education, ethnicity). Most (10 out of 12, 83%) adjusted for cardiovascular risk factors (e.g., hypertension/BP, diabetes mellitus/blood glucose, hyperlipidemia/blood cholesterol, smoking, body mass index, physical activity) and/or cardiovascular diseases (e.g., myocardial infarction, stroke/TIA, heart failure). Nicolini et al. [74] combined a wider range of physical and mental comorbidities in an additive index.

Two thirds of the studies specifically reported adjusting for medications. The study by Knight et al. [69] provided a comprehensive list of the psychoactive and cardioactive drugs considered. Among the other studies, the most frequently mentioned medications were anti-hypertensives (6 out of 7 studies, 86%) followed by antiarrhythmics (3 out of 7 studies, 43%), cardiac glycosides (2 out 7 studies, 29%), lipid-lowering drugs (2 out of 7 studies, 29%), antidepressants (1 out of 7 studies, 14%) and calcium channel blockers (1 out of 7 studies, 14%). One third of the studies included the apolipoprotein E genotype among the potential confounders. Four studies adjusted for HR [7,67,70,74]. One study also adjusted for the Epworth sleepiness score [75].

It should be noted that since HRV is influenced by HR [3], with the exception of the transformed frequency-domain indices [87] and the HRF metrics [8], it is generally recommended that HRV be corrected for HR [3,88,89]. However, some [90] caution that adjustment may remove meaningful variance in the outcomes of interest that can be attributed to autonomic phenomena.

### 3.6. Study Results

The study results are presented first more generally, in chronological order, for each individual study. They are then grouped in terms of the activity of the specific limbs of the ANS (PNS and SNS) in relation to all cognitive outcomes and in relation to specific cognitive domains. It should be noted that the use of transformed indices (LFn and, especially, LF/HF) as markers of SNS activity has received critique as well as support (see Introduction). The relationship between PNS and SNS activity and the cognitive outcomes is illustrated in Table 3.

#### 3.6.1. General Relationship between HRV and Cognition

Britton et al. [66] investigated a large cohort of middle-aged British civil servants from the Whitehall II study. They evaluated HRV and cognition both at baseline and after a 5-year follow-up. HRV (5 min) was assessed by time- and frequency-domain indices, and cognition by a battery of 5 cognitive tests. There was no relationship between HRV at baseline and cognitive performance at follow-up, but lower SDNN, HF and LF predicted a greater decline in the Mill Hill test after adjustment for demographics (odds ratio (OR) and 95% confidence interval (CI) for being in the worst quintile of change = 1.16, 1.05–1.29 for SDNN, 1.18, 1.06–1.31 for HF and 1.19, 1.08–1.32 for LF).

Mahinrad et al. [67] considered a large cohort of older subjects, with pre-existing vascular disease or with at least one major vascular risk factor, from the PROspective Study of Pravastatin in the Elderly at Risk (PROSPER). They evaluated HRV at baseline, and cognition both at baseline and after an approximately 3-year follow-up. HRV (10 s) was assessed by SDNN and cognition by a battery of 3 cognitive tests. After correction for a number of potential confounders, lower SDNN predicted greater decline in the Letter–Digit Coding test (*p* value for mean difference across thirds of SDNN = 0.038).

Zeki Al Hazzouri et al. [64] examined a large cohort of middle-aged participants from the Coronary Artery Risk Development in Young Adults (CARDIA) study. They evaluated HRV at baseline and cognition after a 5-year follow-up. HRV (10 s) was measured by time-domain indices and cognition by 3 cognitive tests. In fully-adjusted models higher SDNN was associated with better performance on the Stroop test (unstandardized regression coefficient (B) for quartile (Q) 3 versus Q1 SDNN = −1.21, *p* = 0.04; B for Q2 versus Q1 SDNN = −1.72, *p* < 0.01).

Kim et al. [68] recruited a small sample of older neurological outpatients who, at baseline, were diagnosed with Mild Cognitive Impairment (MCI) and underwent HRV analysis. HRV was evaluated in the time and frequency domains. These subjects were then followed up for an average of 30 months until they developed DLB (MCI-DLB group) or AD (MCI-AD group), based on clinical evaluation (and dopamine transporter imaging for DLB). When comparing the MCI-DLB and MCI-AD groups, matched on demographics, the former showed reduced SDNN, RMSSD, TP, LF and HF (also relative to a cognitively normal control group) (*p* value range for mean differences between MCI-DLB and MCI-AD < 0.001–0.046). The authors thus suggested that, in subjects with MCI, HRV may aid in predicting progression to different subtypes of dementia.

Knight et al. [69] analyzed data from a large cohort of middle-aged subjects recruited into the Midlife in the United States (MIDUS) study. They evaluated HRV at baseline, and cognition both at baseline and after an approximately 10-year follow-up. HRV (short-term) was indexed by HF and assessed both in resting conditions and in response to a psychological (cognitive task) and physical (active standing) challenge. Cognition was assessed by a telephone-administered battery of 6 cognitive tests whose summary score was also decomposed into an episodic memory and an executive functioning factor. While resting HF was only marginally linked to change in cognition, a greater PNS responsivity to the cognitive challenge (in terms of PNS recovery and reactivity) predicted an attenuated decline in cognitive functioning after controlling for covariates (B = −0.176, *p* = 0.007 for recovery and B = 0.192, *p* = 0.007 for reactivity). This finding was more prominent for the executive functioning factor (B = −0.248, *p* < 0.001 for recovery and B = 0.263, *p* < 0.001 for reactivity) than for the episodic memory factor (B = −0.149, *p* = 0.142 for recovery and B = 0.234, *p* = 0.026 for reactivity) and was limited to individuals with low levels of SNS activity as measured by 12-h urinary epinephrine. The response to the orthostatic challenge did not predict change in cognition.

Schaich et al. [70] investigated a large cohort of middle-aged to older subjects enrolled in the Multi-Ethnic Study of Atherosclerosis (MESA). They evaluated HRV at baseline and after a 10-year follow-up, and cognition at follow-up. HRV (10 s) was measured by time-domain indices and cognition was assessed by 3 cognitive tests. After adjustment for confounders, higher SDNN was associated with higher scores on the Cognitive Abilities Screening Instrument (CASI) and Digit Symbol Coding test (DSC) (B = 0.37, *p* = 0.018 and B = 0.80, *p* = 0.013 respectively). There was no connection between change in HRV and any of the cognitive test scores.

Costa et al. [7] considered a large sample of older subjects from the MESA–Sleep cohort and evaluated HRV at baseline, and cognition both at baseline and about 6 years later. HRV, derived from the ECG channel of a polysomnographic recording, was quantified by 3 novel HRF metrics alongside traditional time- and frequency-domain indices. Cognitive performance was assessed by 4 cognitive tests. After adjustment for confounders, greater HRF was associated, for all tests (CASI, DSC, Digit Span forward and backward), with worse cognitive performance at follow-up as (standardized regression coefficient (β) range = −1.54–1.46, *p* value range = 0.003–0.092), as well as steeper cognitive decline from baseline to follow-up (β range = −1.06–1.01, *p* value range = 0.008–0.079). The traditional HRV indices displayed no such associations.

Weinstein et al. [71] studied a large sample of middle-aged to older adults from the Framingham Offspring Cohort who underwent HRV assessment at baseline, were free of dementia at the start of the follow-up, and were followed up for around 10 years. HRV (2 h) was evaluated by time-domain indices and the incidence of all-cause dementia was tracked by a multi-stage surveillance program including clinical interviews, Mini Mental State Examination (MMSE) screening, neuropsychological and neurological examinations and neuroimaging. HRV was not associated with dementia risk across the whole cohort. However, after adjustment for confounders, lower SDNN and RMSSD predicted a higher incidence of dementia in older individuals (i.e., those aged 60 or more at baseline) (hazard ratio (HR) and 95% CI = 0.61, 0.38–0.99 for SDNN and 0.34, 0.15–0.74 for RMSSD).

Chou et al. [72] analyzed data from a large sample of middle-aged to older participants enrolled in the Tainan study. They were dementia-free at follow-up inception and were followed up for about 16 years. HRV (5 min) was assessed with time- and frequency- domain indices, and the occurrence of all-cause dementia was identified by linkage to a national health insurance database. After accounting for a number of covariates, lower SDNN and higher LF/HF ratio were associated with a greater risk of dementia (HR and 95% CI for Q1-Q3 versus Q4 SDNN = 3.23, 1.55–6.73; HR and 95% CI for Q2-Q4 versus Q1 LF/HF = 2.05, 1.12–3.72).

Gafni et al. [73] considered a large sample of middle-aged adults from the CARDIA study. They evaluated HRV at baseline and cognition after a 10-year follow-up. HRV (10 s) was quantified by time-domain indices and cognition by a battery of 6 cognitive tests. After adjustment for confounders, higher SDNN and RMSSD were associated with better performance on the category fluency test (B = 0.40, *p* = 0.029 and B = 0.34, *p* = 0.05 respectively). Additionally, the study modeled the trajectory of physical activity over the 20 years preceding the HRV assessment (i.e., from young adulthood to midlife) and found that HRV indices mediated the association between higher physical activity and better category fluency.

Nicolini et al. [74] recruited a small sample of older geriatric outpatients diagnosed with MCI or normal cognition (NC) at baseline. HRV was evaluated at baseline, and cognition was evaluated both at baseline and after an approximately 3-year follow-up. HRV (5 min) was assessed in resting conditions as well as in response to a sympathetic (active standing) and parasympathetic (paced breathing at 12 breaths/min) challenge. The main focus of the study were transformed frequency-domain indices, but absolute frequency-domain and time-domain measures were also reported. Cognition was quantified by an extensive cognitive battery including 13 tests. Cognitive change was assessed in the episodic memory and executive functioning domains via composite Z-scores. The analyses were performed separately for each of the two groups. After adjustment for potential confounders, significant results were found only in the MCI group. In particular, a greater response to a sympathetic challenge predicted a greater decline in episodic memory (β = −0.528 and *p* = 0.019 for LFn and β = −0.643 and *p* = 0.001 for LF/HF), whereas a greater response to a parasympathetic challenge predicted a lesser decline in executive functioning (β = −0.716 and *p* < 0.001 for LFn and β = −0.935 and *p* < 0.001 for LF/HF).

Sabil et al. [75] conducted a study on a large number of older subjects from the Pays de la Loire Sleep Cohort study. They were patients with newly diagnosed obstructive sleep apnea and no dementia at baseline who were followed up for approximately 7 years. HRV was obtained from pulse oximetry during a sleep recording and assessed in the time and frequency domains. Dementia ascertainment relied on linkage to a national healthcare database. After adjustment for several potential confounders, higher RMSSD and SDNN were associated with an increased risk of all-cause dementia (HR and 95% CI for Q4 versus Q1 RMSSD = 2.34, 1.11–4.92; HR and 95% CI for Q4 versus Q1 SDNN = 2.21, 1.00–4.82).

#### 3.6.2. Relationship between PNS and SNS Activity and All Cognitive Outcomes

Overall, all studies but one [75] found that higher PNS activity was beneficial to cognition, whether in terms of better tests of global [7,69,70] or domain-specific cognition [7,64,66,67,69,70,73,74] or of lower incidence of dementia [68,71,72]. Only Sabil et al. [75] found that higher PNS activity was associated with a higher incidence of dementia. Of the four studies that investigated SNS activity, two reported no relationship with cognition [68,75], and two noted that higher SNS activity was detrimental to cognition in that it predicted a greater decline in episodic memory [74] or a higher risk of dementia [72].

#### 3.6.3. Relationship between PNS and SNS Activity and Specific Cognitive Domains

##### Executive Functioning

All eight studies focusing on cognitive tests assessed executive functioning as well as PNS activity, and found that higher PNS activity predicted better executive functioning. This was true both of resting parasympathetic HRV [7,64,66,67,70,73] and of the HRV response to a challenge. In particular, as to the latter, a greater response to a parasympathetic (paced breathing) challenge [74] as well as greater parasympathetic responsivity to a cognitive challenge [69] correlated with an attenuated decline in executive functioning. None of these studies investigated the relationship between SNS activity and executive functioning.

##### Episodic Memory

Of the eight studies focusing on cognitive tests, two did not assess episodic memory [7,70]. Of the six that investigated episodic memory, five evaluated its relationship with PNS activity [64,66,67,69,73] while one evaluated its relationship with SNS activity [74]. Four studies found no relationship with HRV, all among the former group [64,66,67,73]. Thus, two studies found an association with HRV. They reported that a greater decline in episodic memory was associated with a greater response to a sympathetic (active standing) challenge [74] and an attenuated parasympathetic reactivity to a cognitive challenge [69].

##### Language

Only two studies evaluated language and both assessed PNS activity. They found a positive association of PNS activity with the Mill Hill test but not category fluency [66], and with category fluency [73] respectively. No study evaluated the relationship between SNS activity and language.

### 3.7. Quality Assessment and Risk of Bias

The included studies were of good quality according to AHRQ standards, with a Newcastle–Ottawa Scale score ranging from 6 to 9 stars (see Supplementary Table S3). In all studies, the exposed and non-exposed cohorts were drawn from the same population, both the exposure (HRV) and the outcome (cognition) were objectively measured, and the duration of the follow-up was adequate (≥2 years) [49,91]. Two studies [66,68] controlled for demographics but not for other confounders. Three studies [64,70,73] did not evaluate the outcome at baseline, thus providing the weakest evidence for a potential causal relationship between HRV and cognition [55]. Four studies [67,68,74,75] focused on selected groups at high risk of cognitive decline which, although clinically relevant, are poorly representative of the average adult population. In four studies [7,68,69,70], loss to follow-up was high (>20%) [92] or could not be determined.

## 4. Discussion

While cross-sectional studies have established a link between HRV and cognition, longitudinal studies have important clinical implications in terms of the predictive value of HRV for future cognition and of the potential causal relationship between HRV and cognition. To the best of our knowledge, this is the first systematic review to focus on the longitudinal association between HRV and cognition. It included 12 studies in adult individuals, with different HRV measures and cognitive outcomes. Two thirds of the studies were published from 2020 onwards, indicating this is a very rapidly emerging area of research. In the following sections we provide an interpretation of the results, discuss their relevance to clinical practice and address the limitations of the review process and of the reviewed evidence, highlighting directions for future investigation.

### 4.1. Interpretation of the Results

The interpretation of the results is given in the three main sections below. Because of the questioned validity of the transformed HRV indices as markers of SNS activity, the discussion of the findings pertaining to SNS activity should be taken with some caution.

#### 4.1.1. General Relationship between HRV and Cognition

All studies found that HRV was a longitudinal predictor of cognition. This was true across populations (general versus clinical), HRV indices (time- and frequency-domain and HRF) and cognitive outcomes (performance on cognitive tests and incidence of dementia). The general association between HRV and cognition is in line with the cross-sectional literature and the potential underlying mechanisms have been described in the Introduction.

#### 4.1.2. Relationship between PNS and SNS Activity and All Cognitive Outcomes

There was consistent evidence that PNS activity was beneficial to cognition and some evidence that SNS activity was detrimental to cognition. The differential effects of the PNS/SNS can be traced back to their physiological functions. First, the PNS reduces BP [34] and BPV [35], and also produces cerebral vasodilation [37], thus diminishing the likelihood of cerebral hypoperfusion and damage [36,37,38,39]; on the contrary, the SNS increases BP [34] and BPV [35,36], and also produces cerebral vasoconstriction [37]. Second, the PNS exerts an anti-inflammatory effect [40,41], whereas the SNS can have a pro-inflammatory or anti-inflammatory impact depending on the context [40], and inflammation contributes to brain damage [42,43,44] and cognitive impairment [45,46]. These findings are in keeping with the cross-sectional literature in which higher PNS activity has been rather extensively demonstrated to be associated with better cognition [22,23,24,25,26,27,28,29,30], and in which the relationship between SNS activity and cognition has been less investigated and found to be more equivocal, including detrimental [22,28,93], beneficial [94], mixed [23,24] and no effects [25].

Of note, Sabil et al. [75] found that higher PNS activity increased the risk of dementia. This finding is counterintuitive, also by admission of the authors, but it is in line with the results of studies on incident stroke in the same cohort [95] and incident atrial fibrillation in the same [65] or in another [96] cohort. Since there is evidence that PNS overactivation can trigger atrial fibrillation [97], it can be hypothesized that the association between higher PNS activity and dementia may be mediated by atrial fibrillation-related stroke.

#### 4.1.3. Relationship between PNS and SNS Activity and Specific Cognitive Domains

##### Executive Functioning

Higher PNS activity persistently predicted better executive functioning. Such association is rooted in the neurovisceral integration model (NVM). The NVM is a CAN-based conceptual framework proposed by Thayer et al. [31,32] in which the activity of the prefrontal cortex is indexed by parasympathetic HRV. Since the prefrontal cortex is the site of executive functioning [98,99], the final core assumption of the NVM is that there is a positive relationship between parasympathetic HRV and executive functioning. Over the years, an ever-growing wealth of data have provided firm support to the NVM. Structural and functional neuroimaging studies have shown that parasympathetic HRV is linked to the thickness [100,101,102], cerebral blood flow [103] and functional connectivity [104,105] of the prefrontal cortex. Also, an increasing number of cross-sectional studies have reported an association between parasympathetic HRV and executive functioning (for reviews see [22,106]).

However, there are other possible reasons for the preferential association between PNS activity and executive functioning, and they are, again, related to the physiological effects of the PNS. First, the PNS reduces BP [34] and BPV [35], and higher BP and BPV have been demonstrated to specifically target executive functioning [107,108], likely because the frontal lobes are particularly vulnerable to hypoperfusion [109]. Second, the PNS has a systemic anti-inflammatory effect [40,41], and there is some evidence that systemic inflammation can have a selective impact on executive functioning [110,111].

##### Episodic Memory

Findings on the association between HRV and episodic memory were scant and inconsistent. The fact that two thirds of the studies investigating episodic memory were unable to detect an association with HRV could be due to the demographics of the study samples and to the composition of the cognitive batteries employed. In fact, in late life episodic memory impairment is predominant [112], while in mid-life executive deficits are more common [113,114]. Actually, three of these studies [64,66,73] investigated middle-aged populations, and only one [67] considered an older population which was, nevertheless, selected based on its high risk of vascular disease (and hence of executive dysfunction). Also, all cognitive batteries disproportionately gauged executive functioning, and only one study [74] evaluated both verbal and visual components of episodic memory, and this study was indeed one of the two with significant results.

The two studies that found an association between HRV and episodic memory showed discrepancies in their results. Nicolini et al. [74] found that a greater response to a sympathetic (active standing) challenge predicted a greater decline in episodic memory, while Knight et al. [69] found that greater parasympathetic reactivity to a cognitive task predicted a lesser decline in episodic memory, and that the parasympathetic reactivity to a physical (active standing) challenge did not predict change in cognition. Although the two challenges that produced significant results have methodological differences that limit comparability (i.e., physical versus psychological challenge, different HRV indices), they are both stressors that elicit sympathetic activation/parasympathetic withdrawal. Thus, greater sympathetic activation/parasympathetic withdrawal was associated with both a greater [74] and a lesser [69] decline in episodic memory. Also, Knight et al. [69], unlike Nicolini et al. [74], found no correlation between the response to active standing and cognition. However, it should be noted that, in their case, the orthostatic stress was less intense (standing from sitting and not from supine) and there was no true baseline (the baseline was the recovery from the previous cognitive task, implying that PNS activity was lower and thus less susceptible to further reduction).

The paucity and heterogeneity of findings on the longitudinal relationship between HRV and episodic memory reflects that of the cross-sectional literature in which better episodic memory has been found to be associated with lower PNS activity [68,115,116], higher SNS activity [117,118], higher PNS activity/lower SNS activity [119,120], and higher PNS and SNS activity [121]. Although there is no established psychophysiological model linking HRV to episodic memory, there is increasing recognition that components of the CAN other than the prefrontal cortex can underlie the association between HRV and cognition. In particular, Nicolini et al. [74] hypothesized, based on diverse lines of evidence from the literature, that there may exist a “sympathetic” CAN comprising the hippocampus, insula and locus coeruleus, which are involved both in episodic memory and in generating sympathetic outflow. However, they did not find the expected negative association between the response to a sympathetic challenge and decline in episodic memory, but rather a positive one. Further research will therefore be needed to elucidate the likely complex neural underpinnings of the relationship between HRV and episodic memory.

##### Language

The two studies evaluating language found a positive association with PNS activity [66,73]. This is consonant with the sparse cross-sectional literature (e.g., [121,122]), and unsurprising since the category fluency and Mill Hill tests also rely upon executive control [83,84,85,123]. To better clarify the association between HRV and language, it could be useful to employ language tests, like the Boston Naming Test, that would seem to be less dependent on executive functioning [124].

### 4.2. Clinical Implications

There are two main clinical implications to the longitudinal relationship between HRV and cognition. First, cognitive aging has heterogeneous trajectories, be it normative [125,126] or pathological [91,127]. Within such diversity, HRV can have predictive value for future cognition, and can therefore be an important tool for prognostic stratification and for the prioritization of interventions to high-risk groups. Relative to the traditional cognitive biomarkers—neuroimaging and cerebrospinal fluid measures [128]—HRV has a number of practical advantages in that it is simple, non-invasive, inexpensive and widely available and applicable. Although there is an acknowledged connection between parasympathetic HRV and psychophysiological non-cognitive outcomes (i.e., psychosocial functioning) [2,5], its investigation was beyond the scope of this review. However, since executive functioning is associated with emotional regulation and social skills [82], it may be speculated that lower PNS activity, in predicting worse future executive functioning, could also predict worse future psychosocial functioning.

Second, treatments to improve cognition can be pharmacological (in cognitive impairment) or non-pharmacological (in subjects across the cognitive spectrum), but both suffer from limited effectiveness, and, in addition, medications are often associated with adverse effects [51,129]. In this scenario, if HRV plays a causal role in cognitive performance then manipulating HRV could be a promising therapeutic approach to improving cognition. HRV biofeedback is a method of enhancing HRV that has become increasingly popular in recent years in a variety of clinical settings [130,131,132]. Participants learn to breathe slowly at their resonance frequency (between 4.5 and 6.5 breaths/min) in order to maximize power in the respiratory frequency band, which is associated with vagal modulation, and thus potently activate the PNS [130,131,132]. Indeed, HRV biofeedback has been shown to benefit cognitive performance in clinical and non-clinical populations across the lifespan [132,133], including older adults without severe cognitive impairment [134] and patients with major depressive disorder [135].

### 4.3. Limitations of the Review Process

The main limitation of this review is the lack of a quantitative analysis. We decided not to perform a meta-analysis because we believed it would not be appropriate due to the clinical and methodological heterogeneity of the available studies [136,137,138,139]. Clinical heterogeneity included differences in study populations (general versus clinical) and participant demographics [136,137,139]. Yet, the inclusion of clinical populations (i.e., with or at higher risk of cognitive impairment) appeared justified by their clinical relevance and by the acknowledged continuum nature of cognitive aging [140]. Methodological heterogeneity included differences in study design (strictly longitudinal or longitudinal time-lagged), duration of follow-up (i.e., timing of outcome measurement), HRV indices (different indices and different HRV methodology even for the same indices), cognitive outcomes (different outcomes and different tests even within a specific domain) and data analysis (different effect size measures, different treatment of the HRV variables as continuous or categorical, different sets of confounders controlled for) [136,137,138,139]. Clinical and methodological heterogeneity were evaluated a priori based on clinical judgement and not a posteriori based on formal statistical testing (e.g., the I^2^ test) because the latter is unreliable when the number of studies is small [139,141]. Although a systematic review need not contain a meta-analysis [136,137,139] and many systematic reviews are narrative [142,143,144], including some on HRV and cognition (e.g., [22]), we do recognize that meta-analyses provide the highest level of evidence for healthcare decision-making [139]. The current review can therefore be considered to set a broader background for future metanalyses on HRV and cognition in longitudinal studies. Hopefully, these will be facilitated by greater homogeneity across studies resulting from better adherence to HRV methodology guidelines and shared protocols for cognitive assessment, as well as from an increase in the number of longitudinal studies which will enable selection of the more comparable ones.

The choice to include only published and English-language articles may have led to publication [145] and language bias [146]. It is therefore possible that there was an overestimation of the association between HRV and cognition, that important evidence may have been missed, and that there is a limitation to the generalizability of the findings. However, we cannot draw any definitive conclusion on publication bias since, in the absence of a meta-analysis, a funnel plot [147] was not produced.

### 4.4. Limitations of the Reviewed Evidence and Future Directions

The reviewed studies were relatively few and had several limitations that identify different potential areas for future research.

#### 4.4.1. Study Design

A minority of studies (3 out of 12, 25%) were longitudinal time-lagged (i.e., they evaluated HRV at baseline and cognition at follow-up). Although these studies are important in determining the predictive value of HRV for future cognition, they have the weakest design in terms of causal inferences [55]. Of the remaining studies, all evaluated HRV at baseline, and cognition at both baseline and follow-up, and were more apt to address causality. Britton et al. [66] also evaluated HRV at follow-up, but did not include change in HRV in their analyses, so that no study evaluated whether change in cognition co-occurred with change in HRV. Examining if change in cognition associates with change in HRV enables a more comprehensive understanding of the relationship between HRV and cognition [47], and provides stronger evidence for causality [55]. Indeed, some [148,149] argue that longitudinal studies should involve at least three repeated measurements of both the exposure and outcome variables so as to more accurately model their relationship. Therefore, future studies should concurrently assess HRV and cognition at both baseline and follow-up and, possibly, even at multiple time-points.

Few studies (2 out 12, 17%) did not control for multiple confounders, and this is a major issue in longitudinal studies given their potential to assess causality [76,136]. Hence, all studies should measure and adjust for a full set of such third variables.

In one quarter of the studies, the loss to follow-up was above the 20% threshold recommended for longitudinal studies, making them vulnerable to attrition bias [92]. It is thus crucial that longitudinal studies implement appropriate retention strategies [92,150].

Half of the studies evaluating the incidence of dementia did not apply a time interval between baseline and the start of the follow-up. Such a wash-out period is important in longitudinal studies with dichotomous outcomes in order to avoid capturing undiagnosed disease, which can distort the association between exposure and outcome via reverse causation bias [151], and should consequently be used.

#### 4.4.2. Study Population

Few studies focused on populations with a baseline diagnosis of cognitive impairment. Kim et al. [68] and Nicolini et al. [74] considered subjects with MCI, no study recruited individuals with dementia. Also, in all studies that evaluated the incidence of dementia but one [68], the outcome was all-cause dementia, and it was not possible to determine whether HRV differentially predicted different types of dementia. Moreover, no study included participants with psychiatric disorders. Therefore, investigation should be extended to subjects at the pathological end of the cognitive spectrum (with incident dementia being differentiated by subtypes) and to subjects with mental illness.

#### 4.4.3. HRV Methodology

All studies but two [69,74] assessed HRV only in resting conditions. It is now largely recognized that the dynamic response to a challenge is a more sensitive measure of autonomic modulation [2,14,16,88]. HRV experimental protocols should increasingly include provocative tests, whether physical (e.g., active standing) or psychological (e.g., cognitive tasks), in order to better detect autonomic dysfunction.

The length of the ECG recordings ranged from 10 s to the duration of night sleep. Such heterogeneity is problematic because it hinders comparability between studies and because there may be specific issues with some recording lengths. In particular, one third of the studies used routine 10-s ECGs and the validity of HRV measures derived from ultra-short-term recordings (<5 min) remains questionable [2,3]. Also, the two sleep studies computed frequency-domain HRV by averaging the values over 5-min epochs, but such averaging, although necessary to the stationarity requirements of power spectral analysis, may obscure detailed information on autonomic modulation [1].

For many studies information was not available on the stabilization period or on artifact-related issues (use of artifact-free data vs. setting a threshold for ectopics, methods of artifact correction). Stabilization is important to ensure steady-state conditions [2], and artifact management influences HRV measures (for instance deletion of ectopics, unlike interpolation, leads to data loss, and to a phase shift to which frequency-domain measures are particularly sensitive) [152].

Thus, studies should conform to the methodological standards of HRV assessment guidelines [1,2]. They recommend a duration of 5 min for short-term recordings and of 24 h for long-term recordings [1,2], preferential processing of the long-term recordings in the time domain [1], a stabilization period of at least 5 min [2] and ectopic correction by interpolation [1].

All studies reported traditional time- and frequency-domain indices, with one study [7] also assessing HRF metrics. As to the frequency-domain indices, two points should be remarked. First, most studies performed the frequency-domain analysis with the FFT/FFT-based methods and one employed the Lomb periodogram. These two methods can provide different results, especially in the presence of systematic patterns in the HR that negatively influence the performance of the Lomb periodogram [153]. Also, no study relied on AR modeling despite there being some evidence [2], not without controversy [1,154], that it may be superior to FFT, and this gap in the literature should be filled by future works. Second, one third of the studies used transformed HRV indices as measures of sympathetic activity. Since this practice has been questioned (e.g., [18,19,20,21]), future studies should also incorporate other HRV and non-HRV indices of sympathetic activity. Among the former is the 0 V% index from the symbolic analysis of HRV, which does not rely on the restrictive assumptions of linearity and reciprocity between the two autonomic branches [155]. Among the latter, the most widely accepted non-invasive ones are the impedance-derived pre-ejection period (PEP) [15] and the QT interval variability [17].

With regard to the HRF metrics, Costa et al. [7] posited that they may be more sensitive indices of parasympathetic activity than the traditional time- and frequency-domain measures. Their proposal was based on the observation by some studies that, in conditions like aging and cardiovascular disease, in which reduced vagal tone is associated with increased fragmentation of the heart rhythm, the traditional parasympathetic HRV measures may be unchanged or even paradoxically increased (since they are reduced by the reduced vagal tone and increased by the increased fragmentation) [7]. Indeed, in the study by Costa et al. [7] only HRF metrics predicted cognitive decline. However, since traditional parasympathetic HRV indices have been consistently associated with cognition even in older subjects [22], such hypothesis warrants additional investigation.

No study evaluated non-linear HRV (e.g., Poincaré plot parameters, entropy, detrended fluctuation analysis coefficients). Although non-linear HRV has a physiological origin that is still somewhat contended [2], it appears to be better suited to capture the complexity of cardiovascular signals [2,9,10,88] and should be explored as a predictor of cognition in further studies.

Finally, only one study [74] monitored the respiratory rate. The confounding effect of respiration on frequency-domain HRV is a critical but often neglected methodological issue in HRV research and has different implications. First, subjects with a respiratory rate outside the HF band, i.e., < 9 breaths/min (0.15 Hz) or > 24 breaths/min (0.40 Hz), should be excluded from analysis because, in these conditions, HF no longer reflects vagal activity [2,156]. Second, even when the respiratory rate falls within the HF range, there is an inverse relationship between HF and respiratory rate, so there should be no significant differences in respiratory rate between groups, across experimental conditions or within a group (if there are, statistical adjustment is possible but controversial) [2,156]. Although less investigated, an effect of respiratory rate on time-domain HRV has also been reported, albeit less significant [157,158,159]. Overall, it is therefore recommended that respiration be monitored during HRV assessment [2].

#### 4.4.4. Evaluation of Cognition

Only few studies evaluated global cognition. This was probably the case because, in the hypothesis that one ANS branch may have a differential relationship with different domains, a summary measure of cognition would be less suited to capture such association. However, global cognition is clinically relevant because it provides information on overall cognitive functioning and as such it should be more frequently considered.

Cognitive assessment was disproportionately focused on executive functioning. There may be two possible reasons for this. First, executive functioning is a broad construct encompassing a wide range of cognitive processes such as working memory and attention [83,98] and is thus gauged by a large number of cognitive tests. Second, the NVM is highly influential in psychophysiology, and it predicts the association of (parasympathetic) HRV with executive functioning [31,32].

Episodic memory was often assessed, but not extensively (i.e., all studies but one used a single memory test), and findings were incongruent. This is particularly problematic since episodic memory is prominently affected by both normal [112] and pathological [160] aging. Future work will be needed to reconcile such inconsistencies, and it would benefit from cognitive batteries tapping both verbal and visual components of episodic memory.

Language was poorly investigated and visuospatial skills were not explored, calling for additional research into the association between HRV and these cognitive domains.

Only two studies evaluated domain-specific composite scores. There is some evidence, although not unequivocal [161], that they may have advantages in terms of greater reliability and lower floor/ceiling effects [162,163], so they should be increasingly exploited in future studies.

There was heterogeneity in the cognitive tests used across studies. This is likely due to the fact that, in the neuropsychological literature, a large variety of cognitive tests are available and there is no consensus on whether some are more robust than others [81,91,164,165]. In order to ensure better comparability between studies, it is thus important that research groups employ shared protocols for cognitive assessment.

#### 4.4.5. Relationship between PNS and SNS Activity and Cognition

There was a consistent association between higher PNS activity and better cognition, and some association between higher SNS activity and worse cognition. However, due to the relatively small number of studies and to their heterogeneity in terms of HRV indices assessed, no HRV index could be considered a standalone biomarker for future cognition (see Supplementary Table S4). Even if there was some suggestion that higher SDNN was more often associated with better cognition, it is not possible to tell whether this has true clinical relevance or simply stems from the fact that SDNN was the most frequently used index (11 studies out of 12, 92%). In order to investigate the predictive capacity of different HRV indices for future cognition further studies will be needed that evaluate multiple or non-SDNN indices.

#### 4.4.6. Underlying Mechanisms

None of the studies directly addressed the potential mechanisms underlying the longitudinal association between HRV and cognition. This can open the way to a multitude of future works pursuing diverse avenues of research and including different assessments such as the functional neuroimaging of the CAN, BP monitoring and the measurement of inflammatory markers. Hopefully, such multiple lines of evidence will help unravel the likely complex and nuanced relationship between HRV and cognition.

## 5. Conclusions

This systematic review found a longitudinal association between HRV and cognition in adults, across populations, HRV indices and cognitive outcomes. Such link appeared to be differential for the two branches of the ANS and for specific cognitive domains. Higher PNS activity was consistently associated with better cognition, and there was some evidence that higher SNS activity was associated with worse cognition. Higher PNS activity persistently predicted better executive functioning, while data on episodic memory and language were more scant and/or controversial. Our findings support the role of HRV as a biomarker of future cognition and, potentially, as a novel therapeutic target. Further longitudinal research will be needed to better investigate the likely complex relationship between HRV and cognition, and it would benefit from concurrent HRV and cognitive assessments at both baseline and follow-up, rigorous HRV methodology, the inclusion of other HRV/non-HRV sympathetic measures, comprehensive neuropsychological testing and meta-analyses. Also, functional neuroimaging, BP monitoring and inflammatory markers may contribute to unravel the mechanistic underpinnings of this relationship.

## Figures and Tables

**Table 1:** Characteristics of the included studies.

Study	Design	N	Population	Age (Mean, Years)	% Female	Education	% White Ethnicity	Exclusion Criteria (Comorbidities/ Medications)	Follow-Up	Recording Device	Conditions	Length of Recording	HRV Indices	Cognition	Confounders Controlled for ^a^	Results
Britton et al., 2008 [66]	L	5375	General (Whitehall II study: British civil servants)	55	29.1	NR	NR	N	5 years	ECG	Resting (supine)	5 min	Time domain: SDNN Frequency domain: HF, LF (Blackman Tukey algorithm)	Cognitive tests: 20-word free recall, Alice Heim 4-I, Mill Hill, Letter fluency, Category fluency	Age, sex, education	Lower SDNN, HF and LF associated with greater decline in Mill Hill test
Mahinrad et al., 2016 [67]	L	3583	Clinical (PROSPER study: subjects at high risk/with vascular disease enrolled in a RCT of pravastatin)	75.0	53.3	Mean age left school: 15.2 years	NR	Non-sinus rhythm + PROSPER criteria ^b^	3.2 years (mean)	ECG	Resting (supine) Morning	10 s	Time domain: SDNN	Cognitive tests: Stroop, Letter- Digit Coding, Picture– Word Learning	Age, sex, education, SBP, DBP, diabetes mellitus, smoking, BMI, myocardial infarction, stroke/TIA, antihypertensive medications and statins, HR	Lower SDNN associated with greater decline in Letter–Digit Coding test
Zeki Al Hazzouri et al., 2017 [64]	LT	2118	General (CARDIA study: adults recruited from 4 US field centers)	45.3	57.7	Mean education: 15.2 years	57.8	Non-sinus rhythm	5 years	ECG	Resting (supine) Morning, at least 2 h after a light snack, no smoking or intense physical activity for 2 h before the examination ^c^	10 s	Time domain: SDNN, RMSSD	Cognitive tests: RAVLT, Digit Symbol Substitution, Stroop	Age, sex, education, ethnicity, SBP, DBP, diabetes mellitus, smoking, BMI, physical activity, depressive symptoms, myocardial infarction, stroke/TIA, antihypertensive medications	Lower SDNN associated with worse performance on Stroop test
Kim et al., 2018 [68]	L	91 ^d^	Clinical (MCI)	69.6	54.9	Mean education: 8.4 years	NR	Focal brain lesions, multiple lacunar infarctions, diffuse white matter hyperintensity, Parkinson’s disease, diabetes mellitus, cardiac diseases, medications such as beta blockers or thyroxine	30 mos (mean)	ECG	Resting (supine) Morning, between 8:00 and 12:00 a.m., no alcohol or caffeinated beverages after 10:00 p.m. and no smoking 1 hr before the recording	NR	Time domain: SDNN, RMSSD Frequency domain: TP, LF, HF, LF/HF (FFT)	Dementia incidence (AD and DLB)	Age, sex, education	Lower SDNN, RMSSD, TP, LF and HF associated with progression of MCI to DLB
Knight et al., 2020 [69]	L	869	General (MIDUS study: community-dwelling English- speaking US adults)	53.8	57.9	NR	81	N	9.3 years (mean)	ECG	Resting (seated) Psychological challenge: cognitive task Physical challenge: active standing Morning, after a light breakfast with no caffeinated beverages ^c^	10 min (resting) 5 min: challenge and recovery from challenge No recovery for the physical challenge	Frequency domain: HF (FFT ^c^)	Cognitive tests: BTACT summary score, BTACT episodic memory subscore, BTACT executive functioning subscore	Age, sex, high blood pressure, depression, emphysema/ COPD, heart disease, heart murmur, circulation problems, TIA/stroke, medications affecting cardiovascular/ autonomic/ cognitive functioning	Greater PNS responsivity (recovery/reactivity) to a cognitive challenge associated with lesser decline in BTACT summary score (with greater effects for executive functioning than for episodic memory subscore)
Schaich et al., 2020 [70]	LT	3018	General (MESA study: adults recruited from 6 US field centers)	59.1	54.9	More than high school: 68.9%	40.2	Non-sinus rhythm, clinical cardiovascular disease at baseline, dementia medications	10 years	ECG	Resting (supine) Morning, in the fasting state	10 s	Time domain: SDNN, RMSSD	Cognitive tests: CASI, Digit Symbol Coding, Digit Span (Forward and Backward combined)	Age, sex, education, ethnicity, SBP, diabetes mellitus, smoking, BMI, physical activity, depression scale score, myocardial infarction, heart failure, stroke/TIA, antihypertensive and antiarrhythmic medications, ApoE genotype, HR	Lower SDNN associated with worse performance on CASI and Digit Symbol Coding test
Costa et al., 2021 [7]	L	1897	General (MESA sleep study: subjects from MESA exam 5 undergoing PSG)	68	53.9	More than high school: 69.8%	36.1	Pacemaker, atrial fibrillation, prevalent dementia	6.4 years (mean)	ECG (from PSG)	Resting (supine) Night	Night sleep (mdn sleep duration 370 min)	HRF indices: PIP, PNNLS, PNNSS Time domain: SDNN, RMSSD Frequency domain: HF (Lomb periodogram)	Cognitive tests: CASI, Digit Symbol Coding, Digit Span Forward, Digit Span Backward	Age, sex, education, ethnicity, gross family income, SBP, diabetes mellitus, total cholesterol, HDL cholesterol, smoking, physical activity, alcohol consumption, depression, cardiovascular and atrial fibrillation events, antihypertensive/lipid-lowering/ antidepressant medications, ApoE genotype, HR	Higher HRF associated with worse performance and greater decline in all cognitive tests
Weinstein et al., 2021 [71]	L	1581	General (FO study: adult offspring of the original FHS cohort)	55.0	53.6	More than high school: 58.6%	NR	Prevalent dementia, atrial fibrillation and congestive heart failure	10.0 years (mdn) ^e^	ECG	Ambulatory	2 h	Time domain: SDNN, RMSSD	Dementia incidence (all-cause)	Age, sex, education, SBP, diabetes mellitus, smoking, physical activity, antihypertensive/antiarrhythmic/cardiac glycoside medications, ApoE genotype	Lower SDNN and RMSSD associated with higher incidence of dementia
Chou et al., 2022 [72]	L	565	General (Tainan study: adult residents of Tainan city, Taiwan)	48.0	56.1	NR	NR	Prevalent dementia, cerebral vascular accidents, antiarrhythmic treatment	15.8 years (mean) ^f^	ECG	Resting (supine) No smoking, alcohol, coffee and tea on the examination day	5 min	Time domain: SDNN Frequency domain: LF, HF, LF/HF (FFT ^c^)	Dementia incidence (all-cause)	Age, sex, socioeconomic status, SBP, FPG, total cholesterol/ HDL cholesterol ratio, smoking, BMI, physical activity, alcohol consumption, ApoE genotype	Lower SDNN and higher LF/HF associated with higher incidence of dementia
Gafni et al., 2022 [73]	LT	1939	General (CARDIA study: adults recruited from 4 US field centers)	45.2 ^g^	58.0	Mean education: 15.0 to 15.6 years	56.9	N	10 years ^g^	ECG	Resting (supine) Morning at least 2 h after a light snack, no smoking or intense physical activity for 2 hr before the examination ^c^	10 s	Time domain: SDNN, RMSSD	Cognitive tests: MoCA, RAVLT, Digit Symbol Substitution, Stroop, Category fluency, Letter fluency	Age, sex, education, ethnicity, difficulty paying for basics, smoking, high alcohol intake, antihypertensive/ antiarrhythmic/ cardiac glycoside medications	Lower SDNN and RMSSD associated with worse performance on Category fluency test
Nicolini et al., 2022 [74]	L	71 ^h^	Clinical (MCI)	78.2	77.5	Mean education: 11.0 years	NR	Non-sinus rhythm, heart disease, diabetes mellitus, neurological and psychiatric diseases, severe diseases, beta-blockers, alpha-blockers, centrally-acting CCBs, class I and III antiarrhythmic drugs, digoxin, TCAs, SSNRIs, atypical antidepressants, antipsychotics and AChEIs	2.8 years (mean)	ECG	Resting (supine) Physical challenge: active standing and paced breathing at 12 b/min (supine) Morning, between 8:30 and 11:30 a.m., after a light breakfast and no caffeinated beverages, alcohol, smoking and intense physical activity in the 12 h prior to testing	5 min	Time domain: SDNN, RMSSD, pNN50 Frequency domain: LFn, LF/HF, TP, LF, HF (FFT)	Cognitive tests: episodic memory score and executive functioning score	Age, sex, education, physical activity, physical/ mental comorbidity, HR	Greater response to a SNS challenge (LFn and LF/HF) associated with greater decline in episodic memory score, greater response to a PNS challenge (LFn and LF/HF) associated with lesser decline in executive functioning score
Sabil et al., 2022 [75]	L	3283	Clinical (PDL Sleep Cohort study: subjects with obstructive sleep apnea)	69 ^i^	35.4	Less than high school diploma: 70.4%	NR	Prevalent dementia, atrial fibrillation, pacemaker, neuromuscular diseases, chronic respiratory failure	6.8 years (mdn)	Pulse oximeter	Resting (supine) Night	Night sleep (duration NR)	Time domain: SDNN, RMSSD Frequency domain: LFn, HFn, LF/HF (method NA)	Dementia incidence (all-cause)	Age, education, hypertension, alcohol intake, depression, stroke, CCBs, Epworth sleepiness score	Higher RMSSD and SDNN associated with higher incidence of dementia

Legend: ^a^ In fully-adjusted models. ^b^ Includes (not exhaustive): MMSE < 24, history of malignancy, organ transplant recipient, cyclosporin treatment, abnormal laboratory findings (blood count, glucose, liver/kidney/thyroid function). ^c^ Information retrieved from additional references (see Supplementary Table S2). ^d^ n = 23 MCI-DLB, n = 32 MCI-AD, n = 36 NC. ^e^ 14.1 years (mean) between baseline and start of follow-up. ^f^ 5 years between baseline and start of follow-up. ^g^ HRV assessment (Year 20) taken as baseline of the study. ^h^ n = 34 MCI, n = 37 NC. ^i^ Median. Abbreviations: AChEIs, acetylcholinesterase inhibitors; AD, Alzheimer’s disease; ApoE, apolipoprotein E; BMI, body mass index; BTACT, Brief Test of Adult Cognition by Telephone; CARDIA, Coronary Artery Risk Development In Young Adults; CASI, Cognitive Abilities Screening Instrument; CCBs, calcium channel blockers; COPD, chronic obstructive pulmonary disease; DBP, diastolic blood pressure; DLB, dementia with Lewy bodies; ECG, electrocardiogram; FHS, Framingham Heart Study; FFT, fast Fourier transform; FO, Framingham Offspring; FPG, fasting plasma glucose; HDL, high-density lipoprotein; HF, high-frequency power; HFn, normalized HF; HR, heart rate; HRF, heart rate fragmentation; HRV, heart rate variability; L, strictly longitudinal; LF, low-frequency power; LFn, normalized LF; LF/HF, LF to HF ratio; LT, longitudinal time-lagged; MCI, mild cognitive impairment; MESA, Multi-Ethnic Study of Atherosclerosis; MIDUS, midlife in the United States; MMSE, Mini Mental State Examination; MoCA, Montreal Cognitive Assessment; N, no; NA, not available; NC, normal cognition; NN, normal to normal; NR, not reported; PDL, Pays de la Loire; PIP, percentage of inflection points; PNNLS, percentage of ∆ NN intervals in long segments; PNNSS, percentage of ∆ NN intervals in short segments; pNN50, percentage of successive NN intervals that differ by more than 50 ms; PNS, parasympathetic nervous system; PROSPER, Prospective Study of Pravastatin in the Elderly at Risk; PSG, polysomnography; RAVLT, Rey Auditory–Verbal Learning Test; RCT, randomized control trial; RMSSD, root mean square of successive differences of the NN intervals; SBP, systolic blood pressure; SDNN, standard deviation of the NN intervals; SNS, sympathetic nervous system; SSNRIs, selective serotonin–noradrenaline reuptake inhibitors; TCAs, tricyclic antidepressants; TIA, transient ischemic attack; TP, total power.

**Table 2:** Cognitive tests used to evaluate global and domain-specific cognition in the included studies.

Cognition	Cognitive Test	Study
Global cognition	BTACT summary score ^a^	Knight et al., 2020 [69]
	Cognitive Abilities Screening Instrument	Schaich et al., 2020 [70], Costa et al., 2021 [7]
	Montreal Cognitive Assessment	Gafni et al., 2022 [73]
Episodic memory domain	BTACT episodic memory subscore ^b^	Knight et al., 2020 [69]
	Episodic memory score ^c^	Nicolini et al., 2022 [74]
	Picture–Word Learning (visual)	Mahinrad et al., 2016 [67]
	Rey Auditory–Verbal Learning (verbal)	Zeki Al Hazzouri et al., 2017 [64], Gafni et al., 2022 [73]
	20-word free recall (verbal)	Britton et al., 2008 [66]
Executive functioning domain	Alice Heim 4-I	Britton et al., 2008 [66]
	BTACT executive functioning subscore ^d^	Knight et al., 2020 [69]
	Category fluency	Britton et al., 2008 [66], Gafni et al., 2022 [73]
	Digit Span Backward	Schaich et al., 2020 [70], Costa et al., 2021 [7]
	Digit Span Forward	Schaich et al., 2020 [70], Costa et al., 2021 [7]
	Digit Symbol Coding	Schaich et al., 2020 [70], Costa et al., 2021 [7]
	Digit Symbol Substitution	Zeki Al Hazzouri et al., 2017 [64], Gafni et al., 2022 [73]
	Executive functioning score ^e^	Nicolini et al., 2022 [74]
	Letter–Digit Coding	Mahinrad et al., 2016 [67]
	Letter fluency	Gafni et al., 2022 [73], Britton et al., 2008 [66]
	Mill Hill	Britton et al., 2008 [66]
	Stroop	Mahinrad et al., 2016 [67], Zeki Al Hazzouri et al., 2017 [64], Gafni et al., 2022 [73]
Language domain	Category fluency	Britton et al., 2008 [66], Gafni et al., 2022 [73]
	Mill Hill	Britton et al., 2008 [66]

Legend: ^a^ Sum of episodic memory and executive functioning subscores. ^b^ Word List Recall (verbal). ^c^ Prose Recall (verbal) and Rey–Osterrieth Complex Figure-Delayed Recall (visual). ^d^ Digit Span Backward, Category fluency, Number series, Thirty Seconds and Counting Task, Task-Switching. ^e^ Bell Test, Digit Cancellation Test, Digit Span Forward, Digit Span Backward, Trail-Making Test A, Trail-Making Test B, Weigl’s Test, Cognitive Estimates-total, Cognitive Estimates-bizarre, Raven’s Colored Progressive Matrices, Letter fluency. Abbreviations: BTACT, Brief Test of Adult Cognition by Telephone.

**Table 3:** Relationship between ANS activity and cognitive outcomes in the included studies.

		Relationship between PNS Activity and Cognitive Outcome					Relationship between SNS Activity and Cognitive Outcome				
	Cognitive Outcome	GC	EF	EM	LG	Dem	GC	EF	EM	LG	Dem
**Study**	Britton et al., 2008 [66]	NA	+	No	+	NA	NA	NA	NA	NA	NA
	Mahinrad et al., 2016 [67]	NA	+	No	NA	NA	NA	NA	NA	NA	NA
	Zeki Al Hazzouri et al., 2017 [64]	NA	+	No	NA	NA	NA	NA	NA	NA	NA
	Kim et al., 2018 [68]	NA	NA	NA	NA	-	NA	NA	NA	NA	No
	Knight et al., 2020 [69]	+	+	+	NA	NA	NA	NA	NA	NA	NA
	Schaich et al., 2020 [70]	+	+	NA	NA	NA	NA	NA	NA	NA	NA
	Costa et al., 2021 [7]	+	+	NA	NA	NA	NA	NA	NA	NA	NA
	Weinstein et al., 2021 [71]	NA	NA	NA	NA	-	NA	NA	NA	NA	NA
	Chou et al., 2022 [72]	NA	NA	NA	NA	-	NA	NA	NA	NA	+
	Gafni et al., 2022 [73]	No	+	No	+	NA	NA	NA	NA	NA	NA
	Nicolini et al., 2022 [74]	NA	+	NA	NA	NA	NA	NA	-	NA	NA
	Sabil et al., 2022 [75]	NA	NA	NA	NA	+	NA	NA	NA	NA	No

Legend: +, positive association; -, negative association; No, no association. Abbreviations: ANS, autonomic nervous system; PNS, parasympathetic nervous system; SNS, sympathetic nervous system; GC, global cognition; EF, executive functioning domain; EM, episodic memory domain; LG, language domain; Dem, dementia; NA, not assessed. Note: a relationship is considered to be present when at least one of the PNS or SNS indices is associated with the cognitive outcome (and in the case of domain-specific cognition, when the association is at least with one test within the domain).

## Data Availability

No new data were created or analyzed in this study. Data sharing is not applicable to this article.

## Supplementary Materials

The following supporting information can be downloaded at: https://www.mdpi.com/article/10.3390/jcm13010280/s1, Table S1: Further aspects of HRV methodology of the included studies. Table S2: Additional references used to retrieve greater details of HRV methodology of the included studies. Table S3: Quality assessment of the included studies based on the Newcastle-Ottawa Scale for Cohort Studies. Table S4: Relationship between HRV indices and cognitive outcomes in the included studies.
