# Supplementary material for: Heart Rate Variability and Cognition: A Narrative Systematic Review of Longitudinal Studies
Source: J Clin Med. 2024 Jan 4;13(1):280. doi: 10.3390/jcm13010280 (PMC10780278; doi:10.3390/jcm13010280)
Supplement: Supplementary file 1 [file jcm-13-00280-s001.zip › jcm-2729658-supplementary.pdf]

Supplementary Materials

Supplementary Table S1. Further aspects of HRV methodology of the included studies.

Study	Stabilization period	Artifact detection	Artifact correction	Respiratory rate
Britton et al., 2008 [66]	5 min [a]	Fully automated algorithm [a]	Artifact-free data [a]	Not assessed
Mahinrad et al., 2016 [67]	NA	Fully automated algorithm	NA	Not assessed
Zeki Al Hazzouri et al., 2017 [64]	NA	Automated algorithm and visual inspection	NA	Not assessed
Kim et al., 2018 [68]	NR	NR	Artifact-free data	Assessed but not reported
Knight et al., 2020 [69]	NA	Automated algorithm and visual inspection [a]	Inclusion threshold: ectopics < 20 % of total beats [a] (method of correction: interpolation [a])	Assessed [a] but not reported
Schaich et al., 2020 [70]	At least 5 min [a]	Automated algorithm and visual inspection	Inclusion threshold: ectopics ≤ 50 % of total beats [a] (method of correction NA)	Not assessed
Costa et al., 2021 [7]	NA	Automated algorithm and visual inspection	Inclusion threshold: ectopics < 25 % of total beats (method of correction NA)	Assessed but not reported
Weinstein et al., 2021 [71]	NAP	Automated algorithm and visual inspection [a]	Inclusion threshold: ectopics < 10 % of total beats [a] (method of correction NA)	Not assessed
Chou et al., 2022 [72]	At least 15 min	NA	NA	Not assessed
Gafni et al., 2022 [73]	NA	Automated algorithm and visual inspection [a]	NA	Not assessed
Nicolini et al., 2022 [74]	5 min (for active standing) and 10 min (for resting and paced breathing)	Automated algorithm and visual inspection	Inclusion threshold: ectopics ≤ 1 % of total beats (method of correction: interpolation)	Assessed and reported
Sabil et al., 2022 [75]	NA	Fully automated algorithm [a]	Artifact-free data [a]	Assessed but not reported

Legend

[a] Information retrieved from additional references (see Supplementary Table S2). Abbreviations: NA, not available; NAP, not applicable; NR, not reported.

Supplementary Table S2. Additional references used to retrieve greater details of HRV methodology of the included studies.

Study	Additional references
Britton et al., 2008 [66]	Hansen et al., 2019 [56]
Mahinrad et al., 2016 [67]	- [a]
Zeki Al Hazzouri et al., 2017 [64]	Friedman et al., 1988 [57]
Kim et al., 2018 [68]	- [a]
Knight et al., 2020 [69]	Ryff et al., 2022 [58], Nevels et al., 2023 [59]
Schaich et al., 2020 [70]	O'Neal et al., 2016 [60], Habibi et al., 2019 [61]
Costa et al., 2021 [7]	- [a]
Weinstein et al., 2021 [71]	Tsuji et al., 1996 [62]
Chou et al., 2022 [72]	Wu et al., 2008 [63]
Gafni et al., 2022 [73]	Friedman et al., 1988 [57], Zeki Al Hazzouri et al., 2017 [64]
Nicolini et al., 2022 [74]	- [a]
Sabil et al., 2022 [75]	Blanchard et al., 2021 [65]

Legend

[a] Greater details not retrievable.

Supplementary Table S3. Quality assessment of the included studies based on the Newcastle-Ottawa Scale for Cohort Studies.

Study	Selection				Comparability	Outcome			Total score (stars)	Quality† (AHRQ standards)
	1) Representativeness of the exposed cohort	2) Selection of the non-exposed cohort	3) Ascertainment of exposure	4) Demonstration that outcome of interest was not present at start of study	1) Comparability of cohorts on the basis of design or analysis	1) Assessment of outcome	2) Was follow-up long enough for outcomes to occur	3) Adequacy of follow-up of cohorts		
Britton et al., 2008 [66]	* (b)	* (a)	* (a)	* (a)	* (a)	* (a)	* (a)	* (b)	8	Good
Mahinrad et al., 2016 [67]	(c)	* (a)	* (a)	* (a)	* (a) * (b)	* (a)	* (a)	* (b)	8	Good
Zeki Al Hazzouri et al., 2017 [64]	* (a)	* (a)	* (a)	(b)	* (a) * (b)	* (a)	* (a)	*(b)	8	Good
Kim et al., 2018 [68]	(c)	* (a)	* (a)	* (a)	* (a)	* (a)	* (a)	(d)	6	Good
Knight et al., 2020 [69]	* (a)	* (a)	* (a)	* (a)	* (a) * (b)	* (a)	* (a)	(c)	8	Good
Schaich et al., 2020 [70]	* (b)	* (a)	* (a)	(b)	* (a) *(b)	* (a)	* (a)	(c)	7	Good
Costa et al., 2021 [7]	* (a)	* (a)	* (a)	* (a)	* (a) * (b)	* (a)	* (a)	(c)	8	Good
Weinstein et al., 2021 [71]	* (a)	* (a)	* (a)	* (a)	* (a) * (b)	* (a)	* (a)	* (b)	9	Good
Chou et al., 2022 [72]	* (a)	* (a)	* (a)	* (a)	* (a) * (b)	* (b)	* (a)	* (a)	9	Good
Gafni et al., 2022 [73]	* (a)	* (a)	* (a)	(b)	* (a) * (b)	* (a)	* (a)	* (b)	8	Good
Nicolini et al., 2022 [74]	(c)	* (a)	* (a)	* (a)	* (a) * (b)	* (a)	* (a)	* (b)	8	Good
Sabil et al., 2022 [75]	(c)	* (a)	* (a)	* (a)	* (a) * (b)	* (b)	* (a)	* (a)	8	Good

Selection

1) Representativeness of the exposed cohort: a) truly representative of the average adult population in the community *; b) somewhat representative of the average adult population in the community *; c) selected group of users e.g. nurses, volunteers; d) no description of the derivation of the cohort.

2) Selection of the non-exposed cohort: a) drawn from the same community as the exposed cohort *; b) drawn from a different source; c) no description of the derivation of the non-exposed cohort.

3) Ascertainment of exposure: a) secure record (e.g. surgical records) *; b) structured interview *; c) written self-report ; d) no description.

4) Demonstration that outcome of interest was not present at start of study: a) yes *; b) no.

Comparability

1) Comparability of cohorts on the basis of the design or analysis: a) study controls for demographics *; b) study controls for additional factors *.

Outcome

1) Assessment of outcome: a) independent blind assessment *; b) record linkage *; c) self-report; d) no description.

2) Was follow-up long enough for outcomes to occur: a) yes (≥ 2 years) *‡; b) no.

3) Adequacy of follow-up of cohorts: a) complete follow-up - all subjects accounted for *; b) subjects lost to follow-up unlikely to introduce bias - number lost ≤ 20 %¶ or description provided of those lost suggested no different from those followed *; c) follow-up rate < 80 %¶ and no description of those lost; d) no statement.

Note: A study can be awarded a maximum of one star for each numbered item within the Selection and Outcome categories. A maximum of two stars can be given for Comparability.

Legend

† Thresholds for converting the Newcastle-Ottawa Scale to AHRQ standards (good, fair, and poor): Good quality: 3 or 4 stars in selection domain AND 1 or 2 stars in comparability domain AND 2 or 3 stars in outcome/exposure domain; Fair quality: 2 stars in selection domain AND 1 or 2 stars in comparability domain AND 2 or 3 stars in outcome/exposure domain; Poor quality: 0 or 1 star in selection domain OR 0 stars in comparability domain OR 0 or 1 stars in outcome/exposure domain.
‡ Adequate duration of follow-up set at ≥ 2 years based on the shortest follow-up of studies investigating the longitudinal trajectories of cognitive function in a general adult population [49] and on the mean follow-up of studies on conversion of MCI to dementia [91].
¶ Acceptable loss to follow-up defined as ≤ 20 % according to a general consensus [92].

Abbreviations: AHRQ, Agency for Health Research and Quality; MCI, Mild Cognitive Impairment.

Supplementary Table S4. Relationship between HRV indices and cognitive outcomes in the included studies.

		**Cognitive outcome**														
		Global cognition			Executive functioning domain			Episodic memory domain			Language domain			Dementia		
**Relationship**		Positive	Negative	No	Positive	Negative	No	Positive	Negative	No	Positive	Negative	No	Positive	Negative	No
**HRV index**	SDNN	Schaich et al., 2020 [70]	X	Costa et al., 2021 [7], Gafni et al., 2022 [73]	Britton et al., 2008 [66], Mahinrad et al., 2016 [67], Zeki al Hazzouri et al., 2017 [64], Schaich et al., 2020 [70], Gafni et al., 2022 [73]	X	Costa et al., 2021 [7], Nicolini et al., 2022 [74]	X	X	Britton et al., 2008 [66], Mahinrad et al., 2016 [67], Zeki al Hazzouri et al., 2017 [64], Gafni et al., 2022 [73], Nicolini et al., 2022 [74]	Britton et al., 2008 [66], Gafni et al., 2022 [73]	X	X	Sabil et al., 2022 [75]	Kim et al., 2018 [68], Weinstein et al., 2021 [71], Chou et al., 2022 [72]	X
	RMSSD	X	X	Schaich et al., 2020 [70], Costa et al., 2021 [7], Gafni et al., 2022 [73]	Gafni et al., 2022 [73]	X	Zeki al Hazzouri et al., 2017 [64], Schaich et al., 2020 [70], Costa et al., 2021 [7], Nicolini et al., 2022 [74]	X	X	Hazzouri et al., 2017 [64], Gafni et al., 2022 [73], Nicolini et al., 2022 [74]	Gafni et al., 2022 [73]	X	X	Sabil et al., 2022 [75]	Kim et al., 2018 [68], Weinstein et al., 2021 [71]	X
	pNN50	X	X	X	X	X	Nicolini et al., 2022 [74]	X	X	Nicolini et al., 2022 [74]	X	X	X	X	X	X
	TP	X	X	X	X	X	Nicolini et al., 2022 [74]	X	X	Nicolini et al., 2022 [74]	X	X	X	X	Kim et al., 2018 [68]	X
	LF	X	X	X	Britton et al., 2008 [66]	X	Nicolini et al., 2022 [74]	X	X	Britton et al., 2008 [66], Nicolini et al., 2022 [74]	Britton et al., 2008 [66]	X	X	X	Kim et al., 2018 [68]	Chou et al., 2022 [72]
	HF	Knight et al., 2020 [69]	X	Costa et al., 2021 [7]	Britton et al., 2008 [66], Knight et al., 2020 [69]	X	Costa et al., 2021 [7], Nicolini et al., 2022 [74]	Knight et al., 2020 [69]	X	Britton et al., 2008 [66], Nicolini et al., 2022 [74]	Britton et al., 2008 [66]	X	X	X	Kim et al., 2018 [68]	Chou et al., 2022 [72]
	LFn [a]	X	X	X	X	Nicolini et al., 2022 [74]	X	X	Nicolini et al., 2022 [74]	X	X	X	X	X	X	Sabil et al., 2022 [75]
	HFn	X	X	X	X	X	X	X	X	X	X	X	X	X	X	Sabil et al., 2022 [75]
	LF/HF [a]	X	X	X	X	Nicolini et al., 2022 [74]	X	X	Nicolini et al., 2022 [74]	X	X	X	X	Chou et al., 2022 [72]	X	Kim et al., 2018 [68], Sabil et al., 2022 [75]

(continued on next page)

	PIP [a]	X	Costa et al., 2021 [7]	X	X	Costa et al., 2021 [7]	X	X	X	X	X	X	X	X	X	X
	PNNLS	Costa et al., 2021 [7]	X	X	Costa et al., 2021 [7]	X	X	X	X	X	X	X	X	X	X	X
	PNNSS [a]	X	Costa et al., 2021 [7]	X	X	Costa et al., 2021 [7]	X	X	X	X	X	X	X	X	X	X

Legend

X, no study. [a] Higher values indicate lower PNS activity. Abbreviations: PNS, parasympathetic nervous system; HRV, heart rate variability; SDNN, standard deviation of the NN intervals; RMSSD, root mean square of successive differences of the NN intervals; pNN50, percentage of successive NN intervals that differ by more than 50 milliseconds; TP, total power; LF, low frequency power; HF, high frequency power; LFn, normalized LF; HFn, normalized HF; LF/HF, LF to HF ratio; PIP, percentage of inflection points; PNNLS, percentage of Δ NN intervals in long segments; PNNSS, percentage of Δ NN intervals in short segments. Note: for the cognitive domains a relationship is considered to be present if the index is associated with at least one test within the domain.
